# Competing mortality risks: predicted cardiovascular disease risk versus predicted risk of breast cancer mortality in patients receiving adjuvant chemotherapy in a single Irish center

**DOI:** 10.1186/s40959-021-00096-w

**Published:** 2021-02-23

**Authors:** Lisa Prior, Hannah Featherstone, David O’Reilly, Killian Nugent, Marvin Lim, John McCaffrey, Michaela J Higgins, Catherine M. Kelly

**Affiliations:** 1 Department of Medical Oncology, Mater Misericordiae University Hospital, Eccles St, Dublin, D07 R2WY Ireland; 2 UCD (University College Dublin) School of Medicine, Belfield, Dublin 4, Ireland

**Keywords:** Breast cancer, Cardiovascular disease, Cardiovascular risk factor, Risk prevention

## Abstract

**Background:**

Due to advances in care, most women diagnosed with breast cancer do not die from the disease itself. Instead, cardiovascular disease (CVD) remains the most frequent cause of death. Many breast cancer patients are older and have established CVD risk factors. They are at further risk due to exposure to anthracyclines, HER2 targeted agents, endocrine therapy and radiotherapy. In this study, we compared the 10-year predicted risk of breast cancer mortality versus that of cardiovascular (CV) morbidity/mortality in breast cancer patients receiving adjuvant chemotherapy using online predictive risk calculators. Furthermore, we evaluated the predicted outcome of CV risk factor optimisation on their overall CV risk.

**Methods:**

This was a cross sectional study. All patients with resected Stage I-III breast cancer who received adjuvant chemotherapy at our centre from September 2015 to November 2016 were identified. Data recorded included demographics, tumor characteristics, treatments and CV risk factors. To calculate predicted 10-year risk of CVD and impact of lifestyle changes, we used the JBS3 (Joint British Society) online risk calculator. To calculate the predicted 10-year risk of breast cancer mortality, we used the PREDICT calculator. Biostatistical methods included Wilcoxon signed rank test for predicted CVD risk pre and post cardiovascular risk optimization.

**Results:**

We identified 102 patients. Of this cohort, 76 patients were ≥ 50 years & 26 were < 50 years of age. The group had significant baseline cardiovascular risk factors: increased BMI (68 %, *n* = 70), ex-smoking (34 %, *n* = 35), current smoking (13 %, *n* = 13), hypertension (47 %, *n* = 47) and dyslipidemia (57 %). Of the total group, 48 % had a high (> 20 %) and 37 % had a moderate (10–20 %) 10-year predicted breast cancer mortality risk. Regarding 10-year predicted risk of CVD, 11 % and 22 % fell into the high (> 20 %) and moderate (10–20 %) risk categories, respectively. Assuming CV risk factor optimisation, there was a predicted improvement in median 10-year CV risk from 26.5 to 9.9 % (*p* = .005) in the high CVD risk group and from 14.0 to 6.6 % (*p* < .001) in the moderate CVD risk group.

**Conclusions:**

Benefits predicted with a CVD risk intervention model indicates that this should be incorporated into routine breast oncology care.

## Introduction

On a global scale, breast cancer is the most frequently diagnosed cancer in women and the leading cause of cancer related mortality in females [1]. However, survival rates continue to improve in the developed world due to therapeutic advances and the introduction of screening programmes [2, 3]. Consequently, most patients do not die from breast cancer and consistent with the general population, cardiovascular disease (CVD) remains the most common cause of death [4]. Over 80 % of women diagnosed with breast cancer are over fifty years of age [5] and already have both intrinsic and modifiable risk factors for CVD such as age, family history, hypertension, elevated cholesterol, smoking, diabetes and elevated body mass index (BMI) [6]. Furthermore, several breast cancer therapies can lead to cardiotoxicity, especially in those patients with an established CVD risk profile. Anthracyclines are associated with cardiomyopathy (the incidence of clinically symptomatic heart failure is between 2 and 5 % [7]). Trastuzumab can induce reversible myocardial dysfunction [8]. Weight gain, which can predispose to CVD, is observed in most breast cancer patients undergoing adjuvant chemotherapy. This is likely attributable to multiple factors including reduced physical activity due to fatigue and premature ovarian failure in premenopausal patients [9]. Breast radiotherapy (particularly left sided) can accelerate coronary atherosclerosis, as well as cardiomyopathy and valvular dysfunction [10]. Aromatase inhibitors have been recently linked with endothelial dysfunction which is an adverse predictor of CVD [11]. Although this has not yet been definitively proven, prolonged ovarian suppression in premenopausal patients may increase the risk of CVD by inhibiting the cardioprotective effects of oestrogen [12]. Overall, CVD appears to be a major competing risk of mortality in breast cancer survivors due to baseline predisposition and the additive cardiotoxic effects of therapy. In this study, we had three objectives. Firstly, we sought to compare the predicted ten-year risk of breast cancer mortality versus the ten-year risk of CVD morbidity and mortality in patients with breast cancer attending our center for adjuvant chemotherapy. Secondly, we sought to identify their predicted lifetime risk of CVD. Finally, we evaluated the impact of cardiovascular risk factor optimisation on their predicted risk of CVD.

## Methodology

We performed a cross sectional study. All patients with resected Stage I-III breast cancer receiving adjuvant chemotherapy at our center from September 2015 to November 2016 were prospectively identified. Informed consent was obtained prior to enrolment. Clinical data was retrieved from patients’ electronic and pharmacy records. Data extracted included information on demographics, medical history, tumor characteristics, proposed adjuvant treatments and cardiovascular risk factors. Systolic blood pressure (SBP) was recorded as an average of two readings taken during adjuvant therapy. Consent was obtained for a serum lipid profile test to be taken at the time of enrolment. Patients were subdivided into 2 categories: under and over 50 years of age. This age threshold was chosen for 2 reasons. Firstly, this is the average age of natural menopause and secondly, we know that the risk of CVD in women dramatically increases after menopause [13]. We elected not to divide by pre and post-menopause status as diagnosis of true menopause can prove complex and non-definitive in a patient group of whom the majority are of perimenopausal age. To calculate predicted ten-year risk of CVD morbidity or mortality, the JBS3 (Joint British Society) online risk calculator was used [14, 15]. This calculator is freely accessible on http://www.jbs3risk.com. There are several CVD risk calculators in use including the Framingham risk score [16]. The JBS3 calculator was chosen as it is validated in a British population which may be more representative of an Irish population compared to a North American cohort. It also incorporates more variables compared to other risk scores. These include age, gender, ethnic group, BMI, deprivation index, smoking status, cholesterol and HDL levels, systolic blood pressure, use of blood pressure medication, diabetes, family history of CVD and other co-morbidities such as chronic kidney disease, atrial fibrillation and rheumatoid arthritis. Furthermore, a predicted lifetime risk can also be generated [15]. This is beneficial in younger patients who may have a low ten-year risk of CVD but a considerable lifetime risk. For lifetime risk, a cut-off age of 83 years was used as this was the average life expectancy of a female in Ireland at the time of study [17]. Of note we used the Pobal HP Deprivation Index [18] to guide the assignation of a surrogate affluence score for the predictive model. To calculate predicted ten-year risk of breast cancer mortality, we used the Predict Calculator [19] which is freely accessible on https://breast.predict.nhs.uk. It has been validated in large prospective studies [20–22] and is endorsed by the American Joint Committee on Cancer (AJCC) [19]. Variables included age, menopause status, oestrogen and HER2-receptor status, KI-67 status, tumour size and grade, nodal status, symptoms at diagnosis and proposed adjuvant treatment. We calculated the risk on the assumption that study candidates would complete their adjuvant therapy. To calculate the impact cardiovascular risk factor optimization would have, we recalculated ten-year CVD & lifetime risk using the JBS3 calculator intervention model and assumed the following conditions: cessation of smoking, SBP 120 mm Hg, total cholesterol (TC) 4.9 mmol/L and high-density lipoprotein (HDL) 1.6 mmol/L. We chose these blood pressure and HDL cut-offs as achievement of these targets are associated with a significant reduction in CVD and CV mortality [23, 24]. The target for TC was chosen based on previous recommendations from the European Society of Cardiology (ESC) that a TC < 5 mmol/L should be maintained [25]. This is also in keeping with guidelines from the Health Service Executive (HSE) in Ireland [26]. Updated guidelines from the ESC focus primarily on LDL-C (low density lipoprotein cholesterol) as a target as opposed to total cholesterol [27]. However, LDL-C as a specific parameter is not included within the JBS3 calculator nor is it included within most other CV risk calculators. We divided patients into low (< 10 %), moderate (10–20 %) and high (> 20 %) risk categories for both predicted ten-year CVD & breast cancer mortality risk. Biostatistical methods included Wilcoxon signed rank test for predicted CVD risk pre and post cardiovascular risk optimization. A *p*-value less than 0.05 was considered statistically significant. All statistical analyses were performed using SPSS statistical software version 26.0 package (IBM Corporation, Armonk, NY, USA).

## Results

### Baseline characteristics

Baseline characteristics are depicted in Table 1. We identified 102 patients. Of this cohort, 76 patients were over fifty years of age & 26 patients were under fifty years of age. The median age of all patients was 54 years (31–76). The median age was 43 years of age (31–49) in the < 50 years of age (yr) group and 59 years (50–76) in the ≥ 50 years of age group. The study population was primarily Caucasian (99 %). Nearly three quarters of patients lived in areas of average affluence. 15 % of patients lived in areas with increased levels of deprivation.

**Table 1:** Demographics

	**All patients (*n* = 102)** **%(n)**	**Under 50yrs (*n* = 26)** **%(n)**	**50yrs or over (*n* = 76)** **%(n)**
**Age**			
◦ Median (range) years	54 (31–76)	43 (31–49)	59 (50–76)
**Ethnicity**			
◦ White	98 (100)	96 (25)	99 (75)
◦ Asian	2 (2)	4 (1)	1 (1)
**Deprivation index**			
◦ 1 (Affluent)	2 (2)	8 (2)	0 (0)
◦ 2	11 (11)	15 (4)	9 (7)
◦ 3 (Average)	73 (74)	69 (18)	74 (56)
◦ 4	11 (11)	4 (1)	13 (10)
◦ 5 (Least affluent)	4 (4)	4 (1)	4 (3)

### Tumour characteristics

Tumor characteristics are detailed in Table 2. Over half (52 %) of patients had Stage II breast cancer and 22 % had Stage III disease. 61 % of patients had tumours larger than 2 cm and 52 % were node positive. Nearly two thirds (65 %) of patients had grade 3 pathology. Over one third (35 %) of patients had HER2 positive breast cancer and 5 % had triple negative breast cancer.

**Table 2:** Tumour characteristics

	**All patients (*n* = 102)** **%(n)**	**Under 50yr (*n* = 26)** **%(n)**	**50yrs or over (*n* = 76)** **%(n)**
**Stage**			
◦ 1	28 (28)	31 (8)	26 (20)
◦ 2	51 (52)	50 (13)	51 (39)
◦ 3	22 (22)	19 (5)	22 (17)
**Tumour size**			
◦ <=2 cm	39 (40)	46 (12)	37 (28)
◦ > 2-5 cm	43 (44)	39 (10)	45 (34)
◦ > 5 cm	18 (18)	15 (4)	18 (14)
**No of positive nodes**			
◦ 0	47 (48)	46 (12)	47 (36)
◦ 1–3	33 (34)	39 (10)	32 (24)
◦ 4–9	14 (14)	12 (3)	15 (11)
◦ 10 or more	6 (6)	4 (1)	7 (5)
**Grade**			
◦ 1	1 (1)	0 (0)	1 (1)
◦ 2	34 (35)	19 (5)	40 (30)
◦ 3	65 (66)	81 (21)	59 (45)
**Receptor status**			
◦ ER+/HER2-	60 (61)	65 (17)	58 (44)
◦ ER+/HER2+	29 (30)	23 (6)	32 (24)
◦ ER-/HER2+	6 (6)	4 (1)	7 (5)
◦ Triple negative	5 (5)	8 (2)	4 (3)

### Treatments associated with cardiovascular toxicity

**Fig. 1:**
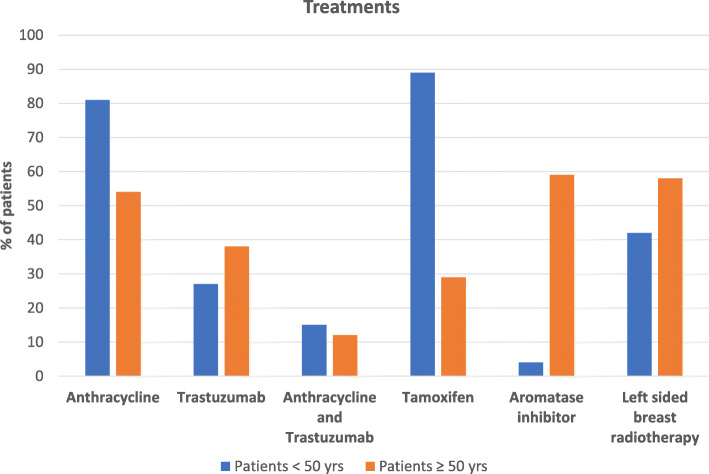
Treatments

Adjuvant treatments associated with cardiovascular toxicity received are depicted in Fig. 1. Anthracycline chemotherapy was administered in 81 % (*n* = 21) of the < 50-yr group and 54 % (*n* = 41) of the ≥ 50-yr group. Trastuzumab was received by 27 % (*n* = 7) of the < 50-yr group and 38 % of (*n* = 29) of the ≥ 50-yr group. The combination of both anthracycline and trastuzumab was received by 15 % (*n* = 4) and 12 % (*n* = 9) of both the < 50 and > 50-yr group, respectively. The majority of the < 50-yr group (89 %, *n* = 23) received Tamoxifen. Over half (59 %, *n* = 45) of patients in the > 50-yr group received an aromatase inhibitor and 29 % (*n* = 22) received Tamoxifen. Left sided radiotherapy was received by 42 % (*n* = 11) and 58 % (*n* = 44) of the < 50-yr and > 50-yr group respectively.

### Cardiovascular risk factors

This patient cohort had significant cardiovascular risk factors at baseline as outlined in Table 3. Nearly 70 % of the total cohort were overweight or obese. Over one third of patients were ex-smokers and 13 % were current smokers. Over half of patients had hypercholesterolaemia (8 of 102 patients did not consent to a lipid profile test). One third of patients had a family history of CVD. Nearly half of patients had hypertension.

**Table 3:** Cardiovascular risk factors

	**All pts (*n* = 102)** **%(n)**	**Pts < 50yrs (*n* = 26)** **%(n)**	**Pts ≥ 50yrs (*n* = 76)** **%(n)**
**BMI**			
◦ 25-25.9 (overweight)	37 (38)	23 (6)	42 (32)
◦ > = 30 (obese)	31 (32)	23 (6)	34 (26)
**Smoking status**			
◦ Ex smoker	34 (35)	39 (10)	33 (25)
◦ Smoker	13 (13)	12 (3)	13 (10)
**Dyslipidemia**			
◦ Total cholesterol > = 5 mmol/L	57 (54/94)	36 (8/22)	64 (46/72)
◦ Median cholesterol (mmol/L)	5.2	4.6	5.4
◦ Statin use	23 (23)	0 (0)	30 (23)
**Hypertension (HTN)**			
◦ Stage I HTN (SBP 130-139 mmHg)	22 (22)	19 (5)	22 (17)
◦ Stage II HTN (SBP > 140 mmHg)	25 (25)	15 (4)	28 (21)
◦ Median blood pressure (mmHg)	128	124	129
◦ Antihypertensive use	19 (19)	8 (2)	22 (17)
**Diabetes mellitus**	3 (3)	0 (0)	4 (3)
**Family history of CVD**	33 (34)	31 (8)	34 (26)

### Ten-year risk of cardiovascular disease versus risk of breast cancer mortality

**Fig. 2:**
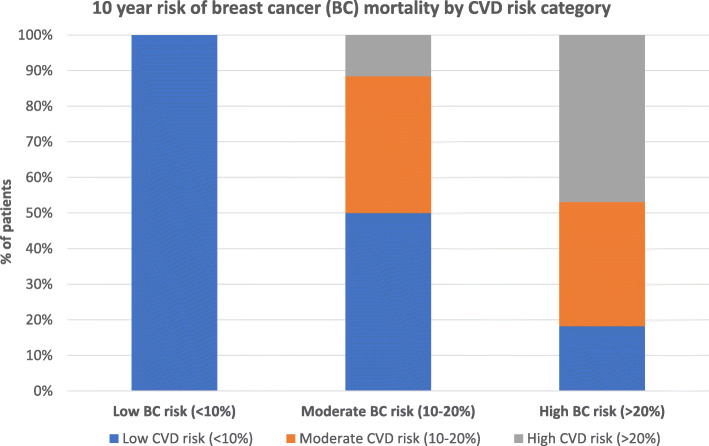
10 year risk of BC mortality by CVD risk category

As aforementioned, 8 of 102 patients (4 patients in each age group) did not consent to a lipid profile test and were excluded from predicted CVD risk versus breast cancer mortality analyses, the results of which are outlined in Table 4. Among the patients in the ≥ 50-yr group, 57 % (*n* = 41), 29 % (*n* = 21) and 14 % (*n* = 10) were considered to have a low (< 10 %), moderate (10–20 %) and high predicted ten-year risk (> 20 %) of cardiovascular disease, respectively. All the patients (*n* = 22) in the < 50-yr age group had only a low predicted ten-year risk of cardiovascular disease. Despite this, the group’s median predicted lifetime risk was high at 50 %. The ten-year predicted risk of breast cancer mortality divided into low, moderate and high-risk categories among the > 50-yr group was 13 % (*n* = 9), 33 % (*n* = 24) and 54 % (*n* = 39) respectively. Among the < 50 year group it was 23 % (*n* = 5), 50 % (*n* = 11) and 27 % (*n* = 6) respectively. Ten percent of patients (*n* = 7) in the > 50 year group had a predicted ten-year risk of cardiovascular disease that exceeded their predicted breast cancer mortality risk. There was no association between the pathological characteristics of their breast cancer and the likelihood of 10 year CVD risk exceeding breast cancer risk. Most patients who were considered to be at high risk for cardiovascular disease were also deemed to be at high risk of breast cancer mortality (Fig. 2).

**Table 4:** 10 year CVD and BC risk

	**All patients (*n* = 94)** **%(n)**	**Pts < 50yrs (*n* = 22)** **%(n)**	**Pts ≥ 50yrs (*n* = 72)** **%(n)**
**10 year risk of CVD mortality/morbidity**			
Low CV risk (< 10 %)	67 (63)	100 (22)	57 (41)
Moderate CV risk (10–20 %)	22 (21)	0 (0)	29 (21)
High CV risk (> 20 %)	11 (10)	0 (0)	14 (10)
**10 year risk of BC mortality**			
Low BC mortality risk (< 10 %)	15 (14)	23 (5)	13 (9)
Moderate BC mortality risk (10–20 %)	37 (35)	50 (11)	33 (24)
High BC mortality risk (> 20 %)	48 (45)	27 (6)	54 (39)
**10 year CVD risk > BC risk**	7 (7)	0 (0)	10 (7)
**10 year BC risk > CVD risk**	93 (87)	100 (22)	90 (65)

### Benefits predicted with CVD risk intervention

**Fig. 3:**
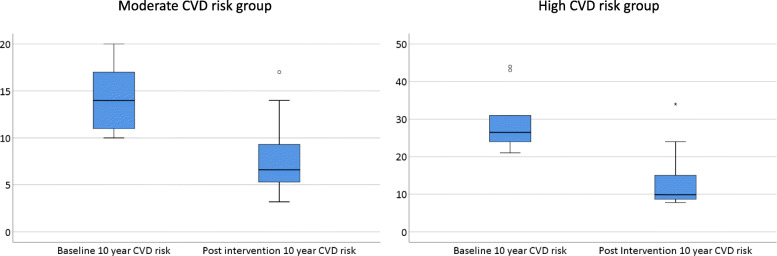
Predicted 10 year CVD risk at baseline and post CV risk factor optimisation in moderate and high CVD risk groups

The benefits predicted with a CVD risk intervention model were noted to be significant and are demonstrated in Fig. 3. Following cardiovascular risk factor optimisation in the high CVD risk group, there was a predicted improvement in the median 10-year CVD risk from 26.5 to 9.9 % (*p* = .005) and in lifetime risk from 47.0 to 38.5 % (*p* = .005). In the moderate CVD risk group, the predicted median 10-year CVD risk post intervention fell from 14.0 to 6.6 % (*p* < .001) and the predicted lifetime risk fell from 61 to 43 % (*p* = < 0.001).

## Discussion

The competing mortality risks of cardiovascular disease and metastatic disease are inextricably intertwined in breast cancer patients receiving adjuvant therapy. Our study demonstrates that patients predicted to be at high risk of breast cancer mortality are also predicted to be at high risk of cardiovascular disease. This study population had significant modifiable cardiovascular risk factors at baseline – over two thirds had a raised BMI and approximately half of patients had hypertension, hypercholesterolemia and a smoking history. Previous population studies have demonstrated a comparable incidence of cardiovascular risk factors among Irish women [28]. Mutual risk factors such as obesity and tobacco use are also associated with an increased incidence of breast cancer. The majority (87 %) of participants within the SWOG breast cancer clinical trials from 1999 to 2011 were noted to have at least one cardiovascular risk factors (64 % had two or more). Furthermore, with each additional cardiovascular risk factor, there was an increased risk of death (HR, 1.23; 95 % CI, 1.08 to 1.40; P = .002) and inferior progression-free survival (HR, 1.12; 95 % CI, 1.00 to 1.25; P = .05) [29].

The combined high and moderate CVD risk groups accounted for a third of our study population. However, the future CV risk of this population may be even higher as predictive CVD risk scores do not incorporate the additive cardiotoxic effect of oncological therapies. A large retrospective cohort study published in 2016 compared the incidence of CVD in survivors of adult-onset cancer to matched non cancer controls (*n* = 73,545). Breast cancer survivors had a CVD incidence rate of 26 % and a higher risk of CVD compared to controls (Incident risk ratio [IR], 1.13; *P* < .01) [30]. A prospective study compared the incidence of CVD events in 1103 breast cancer patients versus controls who had been categorised on enrolment as having a low (< 10 %), intermediate (10–20 %) or high (> 20 %) 10-year risk of developing CVD based on the Framingham risk score. Even in the low-risk group, breast cancer patients had 1.44 (95 % CI 1.00–2.06) times higher risk of a CVD event compared to patients without breast cancer [31].

Our study demonstrated that optimisation of modifiable CV risk factors could lead to a significant predicted decline in 10-year cardiovascular risk, ultimately transferring high and median CV risk patients into a low-risk group. According to the American Heart Association, 80 % of CVD can be prevented through smoking cessation, adherence to a healthy diet, maintenance of a normal weight, engagement in physical activity and optimal blood pressure, diabetic and lipid control [32]. A number of these lifestyle measures are also associated with a decreased risk of breast cancer incidence and recurrence [33–35].

The American Heart Association (AHA) recently released their first scientific statement on the close relationship between CVD and breast cancer. The emergence of the new field of cardio-oncology may address the unmet need with regards to CVD prevention and treatment in cancer patients without compromising cancer survival outcomes. Clinical training programmes, funding and development of clinical guidelines are needed in order to formalise this important sub-speciality. Early identification of patients at risk of CVD is imperative. This can be achieved through standardised recording of CV risk factors and use of validated predictive CVD risk scores in the clinic. Close collaboration between oncology and cardiology is required in patients who are deemed to be at increased risk of CVD. Preventative oncologic strategies such as anthracycline avoidance or use of doxorubicin infusion over bolus can be beneficial in reducing CVD risk. Newer radiation techniques are also associated with a lower risk of complications. Use of biomarkers such as troponin and brain natriuretic peptide as well as newer cardiac imaging techniques such as speckle tracking echocardiography may identify left ventricular strain earlier compared to conventional monitoring. Initiation of beta blockers and ACE inhibitors at the subclinical stage can prevent cardiac remodelling and improve outcomes [12]. Survivorship programmes should incorporate the management of pre-existing modifiable cardiovascular risk factors. Of note, a meta-analysis of 16 prospective studies demonstrated a 48 % reduction in overall mortality (95 % CI 0.42–0.64) and a 28 % reduction in breast cancer mortality (95 % CI 0.60–0.85) in patients with the highest levels of post diagnosis physical activity compared to those with the lowest levels [35]. There is a lack of published data from randomised controlled trials regarding the impact of physical activity on cardiovascular or breast cancer endpoints. However, this is the subject of ongoing trials [12].

There are several limitations with this study. The intervention function of the predictive risk calculator does not measure the impact of weight loss and physical activity on predicted cardiovascular risk. Secondly, lipid profiles were measured while patients were receiving chemotherapy. A recent retrospective study suggests that total cholesterol and LDL levels can increase during chemotherapy while HDL levels can decrease. These changes are transient and lipid levels usually return to baseline 6 months after finishing chemotherapy. While the changes were found to be statistically significant, the numerical change was minimal [36]. Therefore, it is unlikely to have made any impact on the categorisation of true dyslipidemia in this patient cohort. Thirdly, it is recognised that CVD incidence may not be comparable to breast cancer mortality in terms of perceived risk and distant breast cancer recurrence risk may be a better comparator. It is not possible to measure recurrence risk only using the Predict calculator. This was possible using a previous risk calculator Adjuvant Online but this is no longer available for use. In addition, the JBS3 risk calculator does not incorporate the impact of cardiotoxic therapies on the predicted 10-year cardiovascular risk. Finally, the median predicted lifetime risk in the moderate 10-year CVD risk group was noted to be higher than in the high 10-year CVD risk group. This is likely explained by the fact that those in the high CVD risk group are older and have a reduced life expectancy in which to experience a CV event.

## Conclusions

In conclusion, our study demonstrates a significant predicted risk of cardiovascular disease in breast cancer patients receiving chemotherapy. Those at increased risk of CVD are also at a high risk of breast cancer mortality. Meaningful reduction in both median 10 year and lifetime cardiovascular risk is predicted with CV risk factor optimisation in high and moderate CV risk groups. A multidisciplinary approach involving both oncology and cardiology is imperative in mitigating cardiovascular risk and optimising outcomes for breast cancer patients as they navigate through treatment and into the survivorship phase.

## Data Availability

The datasets generated and/or analysed during the current study are not publicly available due to confidentiality but are available from the corresponding author on reasonable request.
